# Decoding visual fatigue in a visual search task selectively manipulated via myopia-correcting lenses

**DOI:** 10.3389/fnins.2024.1307688

**Published:** 2024-04-10

**Authors:** Hyeongsuk Ryu, Uijong Ju, Christian Wallraven

**Affiliations:** ^1^Department of Brain and Cognitive Engineering, Korea University, Seoul, Republic of Korea; ^2^Department of Information Display, Kyunghee University, Seoul, Republic of Korea; ^3^Department of Artificial Intelligence, Korea University, Seoul, Republic of Korea

**Keywords:** children, fMRI, attention, fatigue, myopia

## Abstract

**Introduction:**

Visual fatigue resulting from sustained, high-workload visual activities can significantly impact task performance and general wellbeing. So far, however, little is known about the underlying brain networks of visual fatigue. This study aimed to identify such potential networks using a unique paradigm involving myopia-correcting lenses known to directly modulate subjectively-perceived fatigue levels.

**Methods:**

A sample of *N* = 31 myopia participants [right eye-SE: –3.77D (SD: 2.46); left eye-SE: –3.75D (SD: 2.45)] performed a demanding visual search task with varying difficulty levels, both with and without the lenses, while undergoing fMRI scanning. There were a total of 20 trials, after each of which participants rated the perceived difficulty and their subjective visual fatigue level. We used representational similarity analysis to decode brain regions associated with fatigue and difficulty, analyzing their individual and joint decoding pattern.

**Results and discussion:**

Behavioral results showed correlations between fatigue and difficulty ratings and above all a significant reduction in fatigue levels when wearing the lenses. Imaging results implicated the cuneus, lingual gyrus, middle occipital gyrus (MOG), and declive for joint fatigue and difficulty decoding. Parts of the lingual gyrus were able to selectively decode perceived difficulty. Importantly, a broader network of visual and higher-level association areas showed exclusive decodability of fatigue (culmen, middle temporal gyrus (MTG), parahippocampal gyrus, precentral gyrus, and precuneus). Our findings enhance our understanding of processing within the context of visual search, attention, and mental workload and for the first time demonstrate that it is possible to decode subjectively-perceived visual fatigue during a challenging task from imaging data. Furthermore, the study underscores the potential of myopia-correcting lenses in investigating and modulating fatigue.

## 1 Introduction

The physiological and psychological discomfort experienced following extended engagement in visually demanding tasks is referred to as visual fatigue, sometimes known as eye strain or asthenopia (Dainoff et al., 1981; Bruenech and Kjellevold Haugen, 2007; Tosha et al., 2009). Reading, computer use, and prolonged exposure to digital screens—all of which are increasingly common in today's technology-driven society—are situations that frequently cause eye fatigue (Mocci et al., 2001; Larese Filon et al., 2019). Eye pain, dryness, blurred vision, headaches, but also general mental exhaustion (Takeda et al., 2001; Schakel et al., 2019; Wolkoff, 2020; Zheng et al., 2021) are resultant effects of visual fatigue, which adversely affects productivity and subjective wellbeing.

Despite its ubiquity and impact, the neurological basis of visual fatigue remains poorly understood. The majority of existing studies have mostly concentrated on the effects of physical factors on the ocular system such as excessive accommodation (Sheppard and Wolffsohn, 2018), or eye muscle strain (Richter et al., 2018). In the context of screen use, for example, researchers have suggested the term “digital eye strain” associated with prolonged and continuous use of computer screens due to both increased accommodation demands on the eye muscles, as well as the occurrence of dry eyes as a result of less blinking (Portello et al., 2012). Studies using eye-tracking (Arief et al., 2009) indicated that ocular muscle fatigue can be reflected in both velocity and duration parameters of eye movements. Other studies have used questionnaires (Abdi and Rydberg, 2005), or indirect measures such as critical flicker fusion (CFF) (Lin et al., 2017) to assess physiological aspects of visual fatigue.

Concerning higher-level effects of visual fatigue, recent studies (Csathó et al., 2012; Cao et al., 2014; Rodrigues et al., 2022) utilized a visual attention task to characterize the decline in cognitive performance showing significant adverse effects of fatigue on task performance, particularly when the perceptual load of the target stimuli was high. This phenomenon also relates to the so-called cognitive fatigue—a mental exhaustion that can affect task performance and subjective affective evaluation (Bess and Hornsby, 2014; Karshikoff et al., 2017; Ioannucci et al., 2020; Guillemin et al., 2021). Given that visual fatigue therefore has a much wider-reaching impact on visual and cognitive processing, an investigation of the brain networks triggered by this type of fatigue will be an important, further step in a deeper understanding of this process.

Among the few studies using neuroimaging in the context of visual fatigue, Richter et al. (2018) used Near-Infrared-Spectroscopy (NIRS) to show that oxygenation changes localized around pre-frontal cortex went along in response to increased convergence demands and visual fatigue resulting from repeated vergence and accommodation conditions. In Chen et al. (2017), BOLD-fMRI was employed to investigate cortical areas involved in stereopsis and associated visual fatigue from 3D TV. They found that activity in frontal eye field (BA18) and middle occipital gyrus (BA18/19) was increased under stereoscopic input and suggested that the increased interaction among these brain regions may contribute to visual fatigue when watching 3D video material.

Concerning potential higher-level effects of visual fatigue, Ishii et al. (2014) summarized fMRI studies dealing with cognitive fatigue, suggesting that it is not only caused by impaired activity in task-related brain regions, but is also manipulated by mental facilitation and inhibition systems that modulate the activity of task-related brain regions in order to regulate cognitive task performance. More recently, Bächinger et al. (2019) and Qi et al. (2019) investigated the impact of fatigue on behavioral performance using fMRI, emphasizing the need for a better understanding of the underlying neural mechanisms. In Wylie et al. (2020), a functional connectivity analysis was conducted under increased cognitive workload showing that connectivity in the occipital region of the brain increased with increasing levels of fatigue, indicating the potential usefulness of brain connectome analysis for fatigue detection and classification.

Sustained visual attention tasks, such as visual search represent an effective way to induce and investigate subjective fatigue phenomena. A search task like the popular “Finding Waldo/Wally,” for example, requires substantial amounts of sustained attention, inducing wide-spread neural activation during the attentional phases (Chang et al., 2014; Dehaene-Lambertz et al., 2018). In our previous study (Ryu et al., 2021), we used this task to investigate how fatigue induced by the demanding visual search would be impacted by the wearing of myopia-correcting lenses (so-called Defocus Incorporated Multiple Segments, or DIMS lenses); surprisingly, we found that across both adolescent and adult participants, wearing the DIMS lenses compared to control lenses significantly reduced visual fatigue levels (at similar levels of performance). Specifically, the DIMS lenses showed an average of 23% reduction in fatigue levels compared to the control lenses—a phenomenon that may have physiological origins due to the eye's responses changing with the lens properties (Kollbaum et al., 2019; Kajita et al., 2020), but also due to perceptual and cognitive aspects of spatial attention processes (Lane et al., 2013; Cona and Scarpazza, 2019; De Lestrange-Anginieur et al., 2021).

Given that these myopia-correcting lenses seem to directly affect fatigue, the present study uses this experimental paradigm as an ideal starting point for investigating the underlying neurological changes associated with visual fatigue—specifically, we modulated fatigue during a visual search task when wearing the correcting lenses (“low fatigue”) vs. wearing control lenses (“high fatigue”) in a within-participant design, observing the associated changes in brain activation. Importantly, in the experiments reported here we also dissociate the perceived difficulty of the task from that of perceived subjective fatigue— although these two concepts are correlated (a more difficult search task will most likely also cause more fatigue), it remains to be seen to what degree difficulty and fatigue result in different brain activation. For this, we use multivariate pattern analyses to decode each of these concepts in a brain-wide association study during visual search.

The present work focuses the analysis on an adolescent participant group treated for myopia-progression using the correcting lenses as a first step, as this group is at risk of perceptual and cognitive impairments later in life from myopia (Ong et al., 2013; Megreli et al., 2020), but also as adolescents have prolonged screen exposure leading to potential problems with visual fatigue.

Our work makes the following contributions:

- It is the first to use myopia-correcting lenses as a tool to selectively modulate fatigue within the neuroimaging domain.
- We identify associated brain networks of visual fatigue via a multivariate decoding approach in a visual search task.
- As visual fatigue and perceived task difficulty may go hand-in-hand, we dissociate these two factors in our decoding, highlighting both individually-selective and common brain areas.

## 2 Materials and methods

### 2.1 Participants

The exclusion criteria for recruiting participants were as follows: (1) prismatic glasses (due to an officially diagnosed strabismus), (2) vision correction above the default dispensing range of DIMS lenses necessary (spherical: < –10 Diopter, cylindrical: < –4.00 Diopter), (3) any report of a psychiatric or neurological disease, or other MRI exclusion criteria. We proceeded to recruit a total of 39 Korean middle school students (female: 23; mean age: 13.53 years, SD: 0.25) with myopia. Sample size for the experiment was determined based on our prior study (Ryu et al., 2021) and on similar imaging studies with adolescents on attention research (White et al., 2016; Ladouceur et al., 2018; Shafer et al., 2020). Of the total recruited participants, eight participants had to be excluded due to repeated, excessive head movements (>3 mm, see below) during scanning. For the remaining 31 participants (female: 16; mean age: 13.65 years, SD: 1.05) that were included in the analyses below, their myopia range in spherical equivalent (SE) was for their right eye: −3.77D, SD: 2.46, and for their left eye: −3.75D, SD: 2.45. Our study focused on adolescents who were at risk of developing axial-length-related myopia due to growth, and who wore correction lenses designed specifically for this type of myopia.

The study followed the tenets of the Declaration of Helsinki, and written informed consent was obtained from guardians of the adolescents (middle school students) as well as the students themselves after explanation of the nature and possible consequences of the study. Our study and research protocols were approved by the Internal Review Board (IRB) of Korea University (KUIRB-2019-0310-06).

### 2.2 Lens and frames

**Figure 1:**
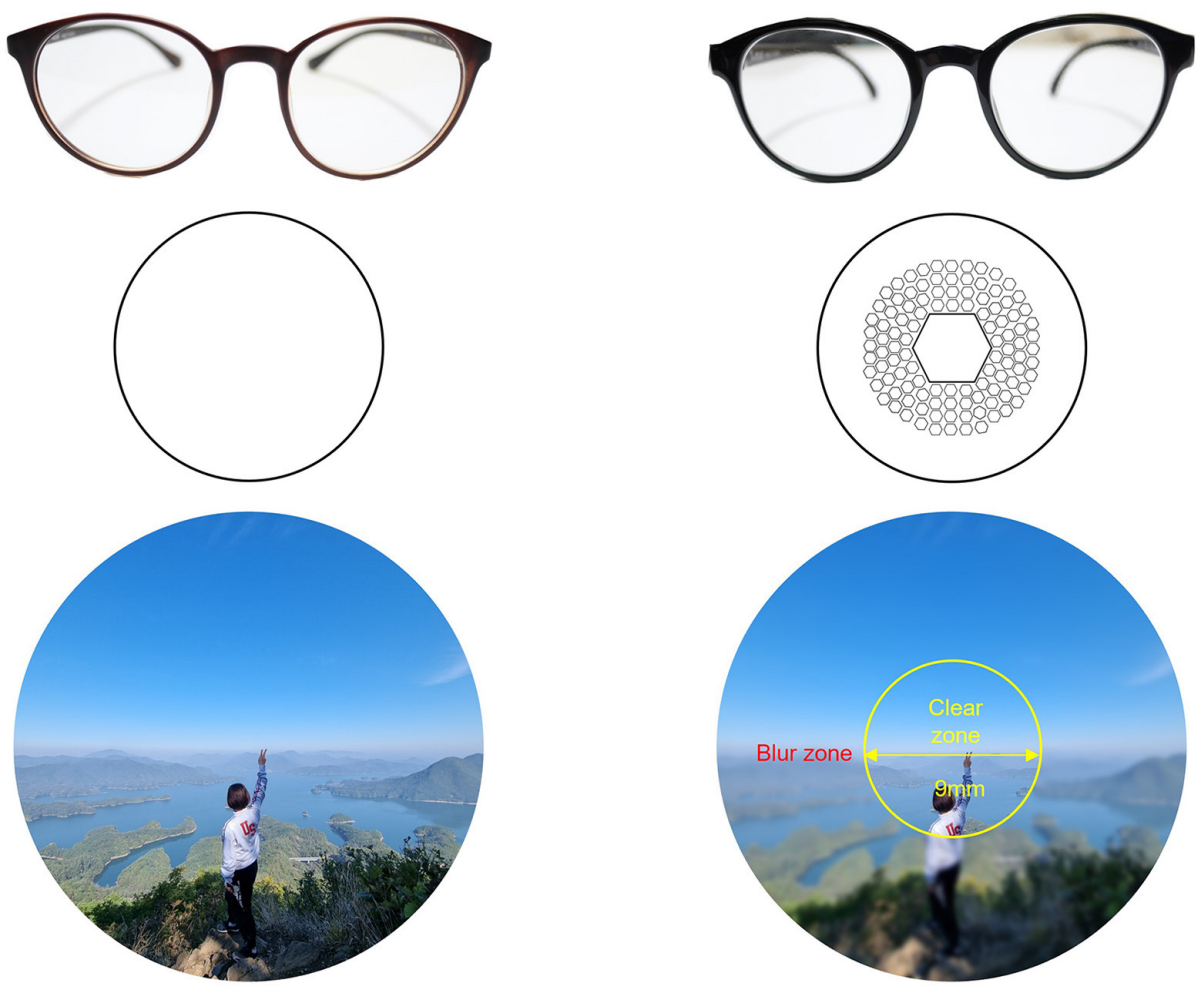
The figure compares single vision (SV) lenses **(left)** vs. defocus incorporated multiple segments [DIMS, **(right)**].

We corrected for each participant's myopia with commercially available, coated clear standard single vision (SV) and Defocus Incorporated Multiple Segments (DIMS) lenses (manufactured by HOYA Vision). The objective of all glasses prescription was to calibrate the refractive error in both eyes to achieve a logMAR0 (equivalent to a desired 20/20; 1.0 vision) with all lenses. If the correction did not reach the desired level, each participant was prescribed maximum correction, with a minimum of logMAR0.1 (20/25; 0.8) respectively. According to the prescription guidelines, the lens powers for SV and DIMS lenses were identical, and there were no observed differences in dizziness or maximal corrected visual acuity attributable to variations in the lenses. Unlike general SV lenses, DIMS lenses have a correct, clear view with only a 9.4 mm limited optical zone in the middle of the glasses, surrounded by a 33 mm defocus zone containing a hexagonal pattern +3.50 D reading spheres, as shown in Figure 1. The different optical zones create a blurred peripheral region spanning roughly 69° × 57° visual angle (horizontal-vertical), excluding a central 13°, non-blurred part. It is this optical change that causes the myopia-correcting effect—for more, see (Lam et al., 2020). To make the spectacles fMRI-safe, non-metal frames were selected and all metal parts from hinge and end-piece areas and from the junction between the front and the temple were removed and replaced with wood.

### 2.3 Stimulus design

We used 20 images of the popular “Finding Wally” illustrations created by the British author Martin Handford as also adopted in our previous behavioral investigation (Ryu et al., 2021). The search task for each image required participants to locate the target (Wally), followed by a difficulty- and fatigue-level-questionnaire after each image. Each of the images was partitioned into a regular 7 × 7 grid indexed by letters (“a–g”) along the *x*-coordinate and by numbers (“1–7”) along the *y*-coordinate. Participants had to identify the grid containing Wally by its corresponding letter/number index.

In selecting the stimuli to be used in the experiment, we tried to include a range of difficulties. The number of individual human figures included inside a grid square could be used as a preliminary estimate of a difficulty level. These numbers were determined for each grid and were used to create a preliminary “Easy” and a “Difficult” category consisting of 10 pictures each. The resulting statistics for the “Easy” category were: Min average (=minimum number of cartoon characters within a grid in the image): 3.1ea (SD = 2.03), Max average (=maximum number of cartoon characters within a grid in the image): 9.3ea (SD = 2.00)—a picture illustrating this category is “FVN and games in ancient Rome.” The resulting statistics for the “Difficult” category were: Min average: 6.6ea (SD = 3.89), Max average: 15.5ea (SD = 6.79)—a picture illustrating this category is titled “The Mighty Fruit Fight.” The difference in this metric was significant for both the minimum and maximum values (*t*-tests, Min: *t*_(18)_ = −2.52, *p* = 0.021, Max: *t*_(18)_ = −2.77, *p* = 0.013). Note, that the experiment itself asks participants for their subjectively perceived difficulty, which will be a dependent variable, and that the values reported above are only given to ensure that our stimuli span a range of potential difficulties.

**Figure 2:**
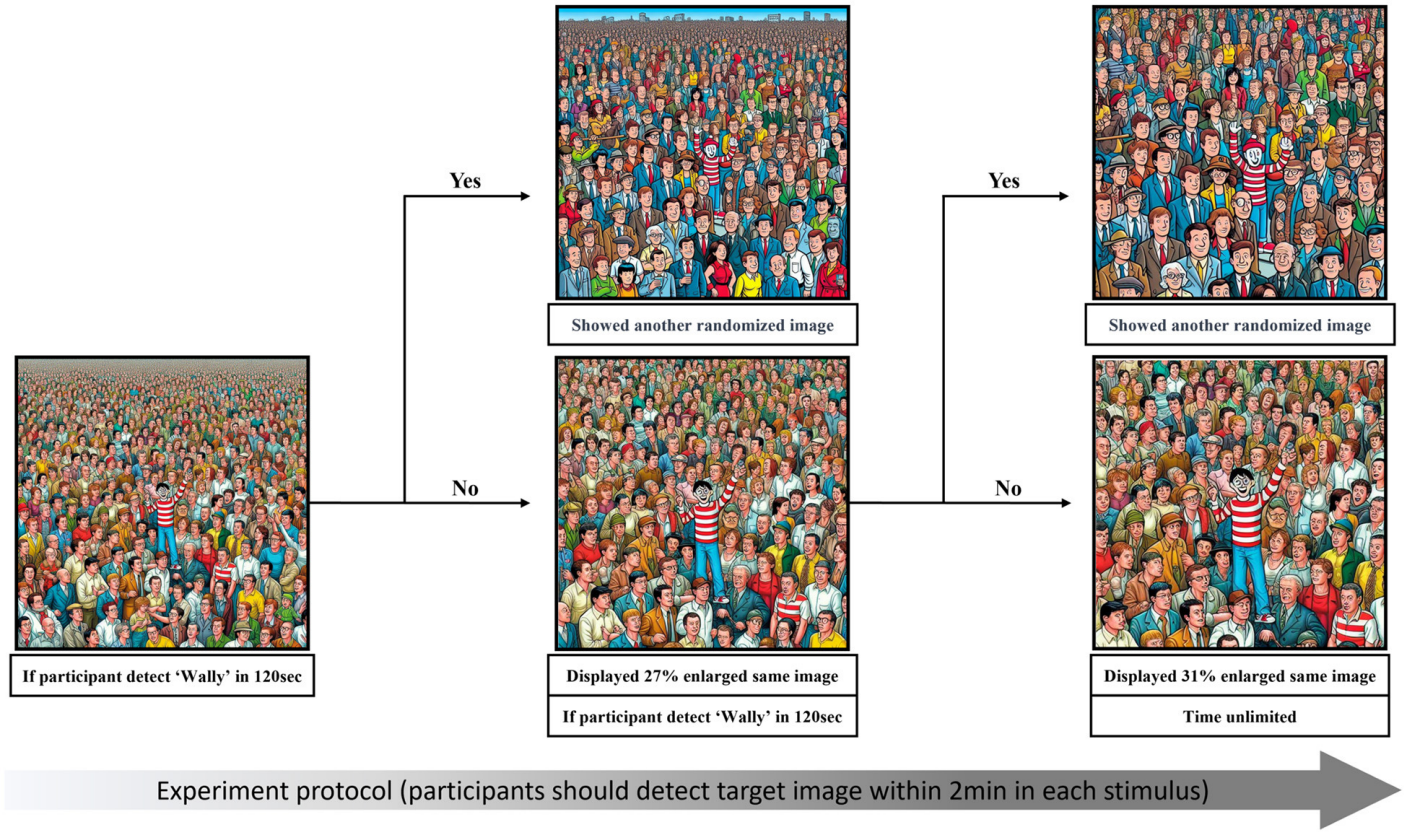
Illustration of the experiment protocol.

To avoid frustration for the adolescent's group, we next created easier versions of each image by enlarging them centered around the true position of Wally in two stages. In the experiment, the second stage was automatically shown if participants did not find Wally for 2 min. This enlarged image was 27% bigger, and showed only 6 × 6 grids. If participants did not find Wally within 2 min, the image was enlarged one more time for the third stage, which showed a 31% larger image with only 5 × 5 grids. Participants were instructed to try to search again in this image, and if they still were not able to find Wally, they were to select any grid square to advance to the next image (see Figure 2).

All stimulus images as well as the interface for the experimental procedure (see below) were generated using Psychtoolbox-3 (3.0.16) for Windows and MATLAB (Mathworks, Natick, USA, 2019a). Stimuli were projected on a 58 × 30 cm (46° × 25°: Horizontal × Vertical); 1,920 × 1,080 pixels at 500 Hz screen by PROPixx (VPixx Technologies, Quebec, Canada).

### 2.4 Procedure

Prior to the scanning experiment, participants underwent an eye examination for determining the proper lens diopters for the DIMS lenses. They were then fitted with the new glasses and a period of adaptation to the new type of lenses Mean: 23.29 days (SD = 14.93)]. During this period, we followed up on how participants were adapting to the DIMS lenses via self-report by calling them after 1,3,5,7, and 14 days and inquiring about any discomfort (such as dizziness or headache) associated with wearing the glasses. In order to participate in the fMRI experiment, we required a minimum of two weeks of adaptation and participants' self-report of no discomfort during wearing of both types of glasses (SV and DIMS)—a criterion that all participants achieved. To ensure consistent levels of fatigue among participants, all individuals were instructed to sleep for a minimum of 6 h before taking part in the experiment. Additionally, we made sure that participants were free of any symptoms of tiredness or fatigue (including headache and drowsiness) of any source prior to the experiment via phone or mail interview. In case of any such symptoms, the experiment was re-scheduled.

Before the scanning session, participants received standard fMRI instructions. Importantly, they were asked not to move their head or body for the duration of the experiment in order to avoid motion artifacts. In addition, they were acquainted with the overall search task by means of a different sample image. Since the experiment also required participants to provide a self-report about subjective fatigue and difficulty levels, we took care to explain the questionnaire items in detail as well. The fatigue questionnaire followed that of our previous study and polled participants' subjective fatigue according to five levels defined as follows: Level 1 = No fatigue: You did not have any problems in locating Wally; Level 2 = A little fatigue: You began to feel a little drained while you sought for Wally; Level 3 = Moderate fatigue: You felt exhaustion while you sought for Wally, but it was possible to persevere in this task; Level 4 = Considerable fatigue: You felt very drained as a result of the visual search task—in addition, may have felt fatigue in your eyes or the urge to yawn sometimes; Level 5 = Severe fatigue: You had difficulty concentrating on the search task, because you felt very fatigued; your eyes may have experienced stronger itching or even pressure, and you may have felt severe urges to yawn. The difficulty levels were defined as the subjective experience of how difficult it was to locate the Wally figure within the (enlarged) images, spanning Level 1 = Very easy; Level 2 = Easy; Level 3 = Moderate; Level 4 = Hard; Level 5 = Impossible (to find Wally).

Participants were next explained how to use the experiment interface, which used two control panels with four right-hand buttons for cursor control, and a left-hand button for clicks. A sample training image was provided to participants without time constraints once they were inside the scanner, such that they were able to test the button control.

In the main experiment, participants were instructed to find Wally for each stimulus shown on the display. If they were unable to locate Wally, the image automatically enlarged to the next version, and again after the next 2 min. Response time (s) was defined as the duration between the onset of stimulus presentation and the participant's selection of the target image (i.e., Wally), and was measured independently for each stimulus presentation. After each Wally stimulus, participants were requested to complete two questionnaires on the screen via button presses, which polled participants' subjective fatigue levels and the perceived difficulty of the preceding image.

There were a total of four blocks with five images, resulting in a total of 20 different Wally images. Between each block, participants were allowed a rest-period of 2 min. The first two blocks (=10 images) used one lens type with participants switching to other type during the middle of the experiment. The order of lens types was counterbalanced across participants; the presentation order of the 20 images was (pseudo-)randomized. Finally, we took care to balance difficulty for each lens type, by selecting both easier and difficult images for each set of 10 images according to the criterion reported above in order to keep up participants' motivation (Ngo, 2015; Olsen et al., 2022).

### 2.5 Data acquisition

MRI data were acquired on a SIEMENS-MAGNETOM Prisma 3T scanner (Siemens Medical Systems, Erlangen, Germany) with a 20-channel Sense head-coil (Center for Neuroscience Imaging Research, Sungkyunkwan University, Suwon, South Korea). Structural MRI images of all participants were collected using a T1-weighted, sagittal high-resolution MPRAGE-sequence [repeat time (TR) = 2,300 ms, echo time (TE) = 2.28 ms, flip angle (FA) = 8 deg, voxel size = 1 × 1 × 1 mm, 192 axial slices]. Functional imaging was performed with a gapless, echo-planar-imaging (EPI) sequence (TR = 2,000 ms, TE = 30 ms, FA = 90 deg, voxel size = 2 × 2 × 2 mm).

### 2.6 Imaging data preprocessing

SPM12 (Wellcome Trust Center for Neuroimaging, London, UK; http://www.fil.ion.ucl.ac.uk/spm/) was used to preprocess the data based on MATLAB (Mathworks, Natick, USA, 2019a). To begin, all scans were realigned to the initial volume, and excessive head translations or rotations were checked. This preprocessing step identified eight participants with excessive head motion (more than 3 mm inter-scan), which therefore were excluded from further analysis.

Following realignment, the T1 image was co-registered with the mean EPI and tissue segmentation was conducted using SPM's New Segment function. Following that, functional data were standardized into MNI space, normalized to 2 × 2 × 2 mm using the DARTEL toolbox normalize function (Ashburner, 2007), and smoothed with an 8 mm FWHM Gaussian kernel.

### 2.7 Behavioral data analysis

The dependent variables consisted of accuracy, response time, as well as the responses to perceived subjective fatigue and perceived subjective difficulty via the questionnaires after each search task—theses were analyzed for the factor of lens type (SV, DIMS) and correlated. For these analyses, we used the non-parametric Wilcoxon Signed-Rank test and correlations in SPSS (Version 25.0, SPSS Inc, Chicago, IL, USA).

### 2.8 fMRI data analysis

Functional MRI data were acquired by 31 participants across four blocks with five images each. The total scanning time was around 1 h and—given that the time to complete one image was variable across participants—the number of acquired volumes spanned a range of volumes as follows: Block 1: mean = 49 volume (16–84 volumes, SD = 32.30), Block 2: mean = 50 volume (16–79 volume, SD = 28.55), Block 3: mean = 40 volume (16–66 volume, SD = 28.14), and Block 4: mean = 55 volume (23–55 volume, SD = 32.70).

#### 2.8.1 Correlational searchlight analysis

A correlational searchlight analysis was conducted aimed at identifying brain regions representing the two measures of subjective fatigue and subjective difficulty as a function of the lens type that was worn in the scanner (SV vs. DIMS lenses). In our study, the use of this analysis was primarily driven by its effectiveness in detecting and localizing complex brain activity patterns across various regions, making it especially useful in uncovering network-based interactions in the brain, which are often obscured in traditional voxel-based methods (Lee Masson et al., 2016; Ju and Wallraven, 2019; Kumar et al., 2020).

For this analysis, first, we extracted standard, uni-variate beta-estimates for each of the 20 images the participants watched, which contrasted one particular image against all other 19 images. This resulted in a 20 × 20 matrix *A*_*p, i*_ for each voxel *i* of participant *p*, which was smoothed by averaging values within a 12 mm radius (216 voxels). We then created matrices containing the differences for a particular image vs. all other images for subjective fatigue (*B*_*p*_) and subjective difficulty (*C*_*p*_). Next, we ran correlations for each participant and voxel between the matrices *A*_*p, i*_ and the matrices B and C, which resulted in 31 correlations for each voxel *i* for each of the two analyses. These 31 correlations were then checked against zero via statistical tests for each voxel, resulting in *p*-value maps for both subjective fatigue and difficulty, respectively. In order to correct for issues arising from multiple comparisons, the *p*-value maps were subjected to a standard false discovery rate (FDR) procedure to determine the final, significant overall correlations between fatigue, difficulty rating, and neural activity over the entire brain. For the searchlight analysis, we used the rsatoolbox 0.1.5 (Nili et al., 2014).

Finally, we used the decoding function of Neurosynth (Yarkoni et al., 2011) to provide a complementary, global interpretation of the correlational searchlight results in the context of its feature terms.

## 3 Results

Participants underwent a scanning session in which several visual search tasks were presented that varied in their difficulty (see Section 2). After each search task, participants were asked to indicate the perceived difficulty and the level of subjectively-experienced visual fatigue. Importantly, fatigue levels were selectively modulated via the use of myopia-correcting lenses (Ryu et al., 2021) with participants completing two sessions in the scanner—one with (the so-called DIMS condition) and one without the lenses wearing standard glasses (the so-called SV condition).

### 3.1 Adaptation period

All participants in the study were myopia patients previously wearing conventional myopia glasses. The new prescriptions we provided closely matched their existing myopic correction statements. Furthermore, according to the DIMS lens fabrication guidelines, the prescription strength for SV lenses was set to be identical to that of myopic correction (see Table 1). However, despite the same prescription power, the adaptation period for DIMS (Mean = 14.87, SD = 5.84) was roughly four times (*W* = 4.87, *p* < 0.001) as long as that for SV (Mean = 3.29, SD = 1.40), indicating increased demands to get used to the different visual input (Kaymak et al., 2022).

**Table 1:** Mean ± SD values for right and left prescription lens power for both SV and DIMS lenses in spherical (Sph), and cylindrical (Cyl).

	**Sph (SD)**	**Cyl (SD)**
Right (OD)	−3.34 D (2.05)	−1.23D (0.68)
Left (OS)	−3.41 D (2.16)	−1.32D (0.64)

### 3.2 Behavioral data analysis

The dependent variables accuracy, perceived fatigue, difficulty, and response time (RT) were first examined for effects of lens type [Single Vision (SV), Defocus Incorporated Multiple Segments (DIMS)]. In order to avoid potential issues of non-normality, we conducted four Wilcoxon Signed-Rank tests for each of the dependent variables—tests were in addition corrected for multiple comparisons with a Bonferroni correction of *n* = 4. Among all variables, we found a main effect only for fatigue—our main measure of interest (*W* = 3.44, *p* < 0.001, see Table 2). This effect showed that the DIMS lenses significantly reduced perceived fatigue on average by 74%. Specifically, the median perceived fatigue scores were 3.50 [interquartile range (IQR): 3.10—3.90] with SV lenses and 2.10 (IQR: 1.70—3.20) using DIMS lenses.

**Table 2:** Mean ± SD, Median with IQR values and Wilcoxon Signed-Rank test results for SV and DIMS lenses.

	**SV**		**DIMS**			
	**Mean (SD)**	**Median (IQR)**	**Mean (SD)**	**Median (IQR)**	**W**	*p* **-value**
Accuracy	0.44 (0.23)	0.50 (0.20–0.60)	0.45 (0.27)	0.40 (0.20–0.60)	0.24	0.81
Fatigue	3.33 (0.85)	3.50 (3.10–3.90)	2.46 (0.98)	2.10 (1.70–3.20)	3.44	< 0.001^***^
Difficulty	3.75 (0.53)	3.60 (3.40–4.10)	3.54 (0.66)	3.40 (3.20–4.10)	1.53	0.13
RT	96.36 (23.34)	98.08 (79.55–112.15)	95.19 (27.42)	96.93 (68.91–116.26)	0.31	0.75

^***^means *p* < 0.001.

**Figure 3:**
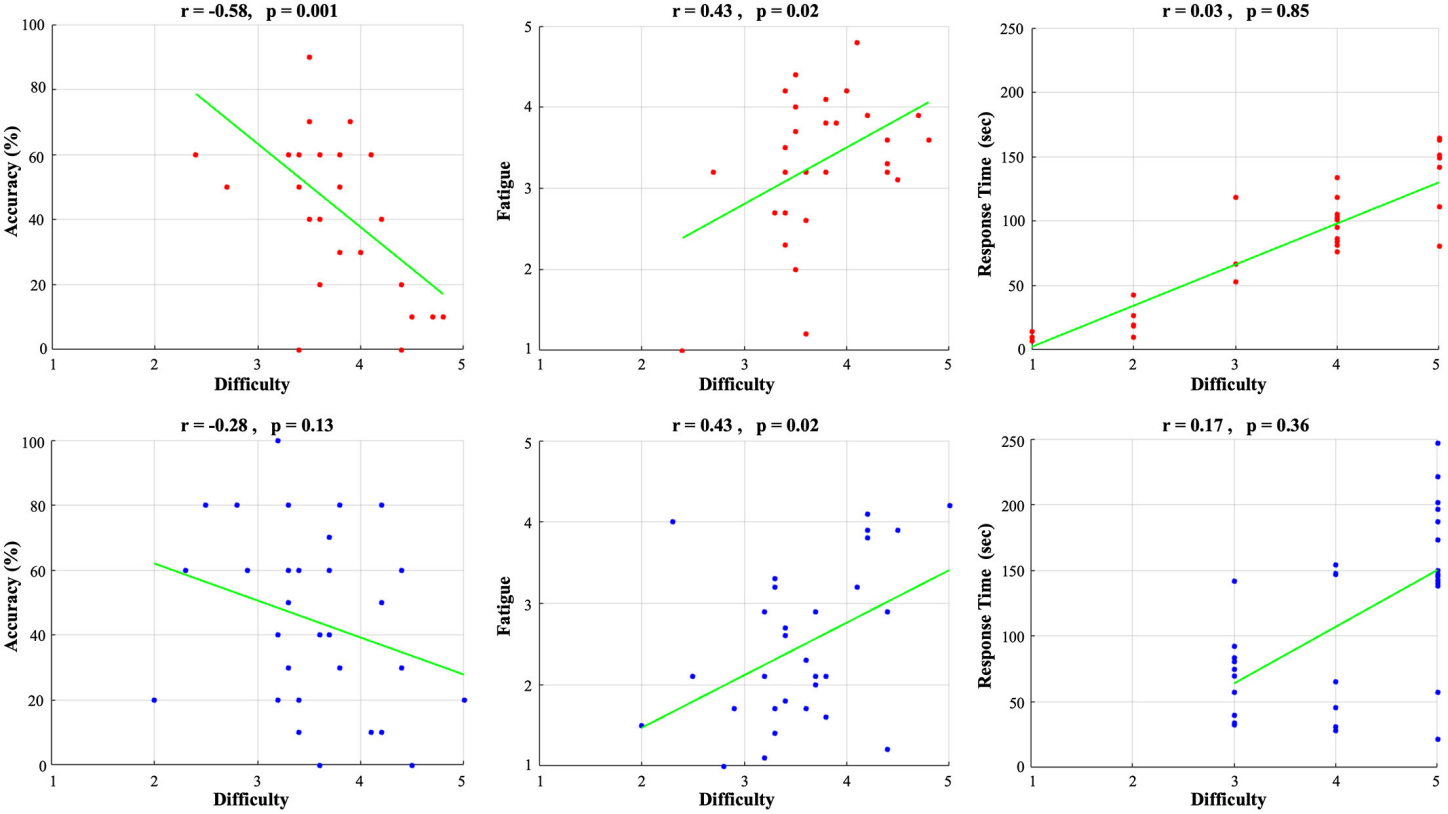
Correlation of subjective difficulty with accuracy **(left)**, subjectively-experienced fatigue **(middle)**, response time **(right)** for SV lenses (upper row, red) and DIMS lenses (lower row, blue).

Next, we looked at potential associations between the measures of accuracy, fatigue, and RT as a function of the subjectively perceived difficulty level via exploratory Pearson correlations (see Figure 3). For both SV and DIMS lenses we found positive correlations of difficulty with fatigue (SV, *r* = 0.43, *p* = 0.02; DIMS, *r* = 0.43, *p* = 0.02) and response time (SV, *r* = 0.03, *p* = 0.85; DIMS, *r* = 0.17, *p* = 0.36), as well as a negative correlation of difficulty with accuracy (SV, *r* = −0.58, *p* = 0.001; DIMS, *r* = −0.28, *p* = 0.13). Importantly, this provides further motivation for our purpose of dissociating the two associated measures of difficulty and fatigue in terms of their neural substrates.

### 3.3 fMRI data analysis

In order to find correlations between neural activity and subjectively-experienced visual fatigue and difficulty, next, we performed a correlational searchlight analysis to identify brain regions that contribute to the decoding of subjective fatigue and difficulty.

**Figure 4:**
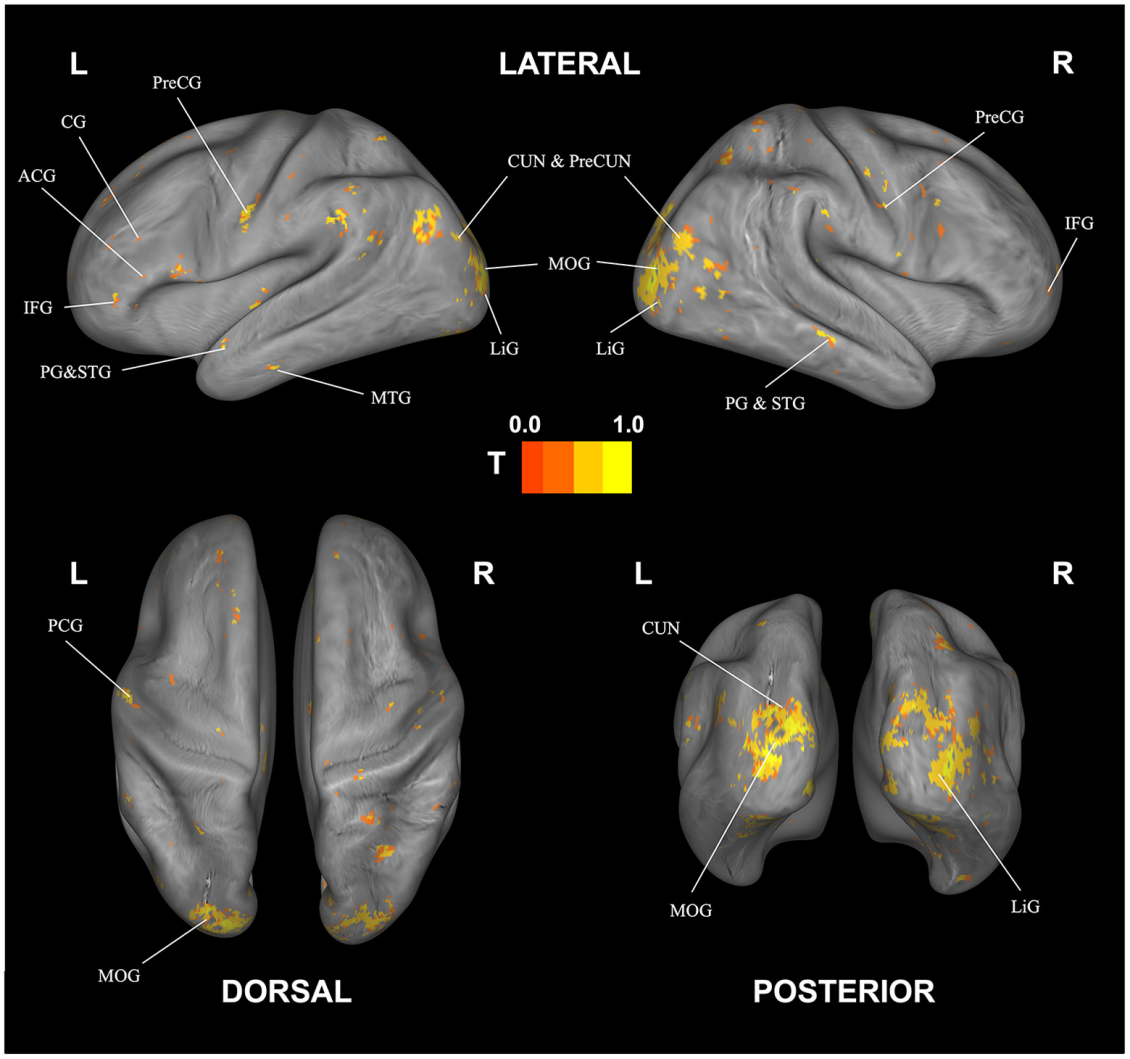
Correlational searchlight analysis results for subjective fatigue. The anatomical labels are abbreviated as follows: ACG, anterior cingulate; CG, cingulate gyrus; CUN, cuneus; IFG, inferior frontal gyrus; LiG, lingual gyrus; MOG, middle occipital gyrus; MTG, middle temporal gyrus; PG, parahippocampal gyrus; PreCG, precentral gyrus; PreCUN, precuneus; STG, superior temporal gyrus. Result was FDR corrected (*p* < 0.05).

Significant, decoding regions with at least 100 voxels for subjective fatigue are listed in Table 3 and visualized in Figure 4. We found a broad network able to decode fatigue including anterior cingulate, cingulate gyrus, culmen, inferior frontal gyrus, middle temporal gyrus, parahippocampal gyrus, precentral gyrus, precuneus, and superior temporal gyrus.

**Table 3:** Searchlight analysis results detailing statistics of the significant regions for subjective fatigue (*p* < 0.05, FDR-corrected, >100 voxels).

**Region (AAL)**	**Peak voxel**	*Z* **-score**	**Number of voxels**
Anterior cingulate	8 42 16	3.97	102
Cingulate gyrus	–2 –32 38	3.78	111
Culmen	32 –58 –28	4.46	201
Cuneus	–16 –94 6	5.46	1,076
Declive	–32 –60 –26	4.65	710
Inferior frontal gyrus	–44 36 0	3.99	102
Lingual gyrus	–24 –76 –8	5.30	1,011
Middle occipital gyrus	28 –84 4	5.53	832
Middle temporal gyrus	42 –70 16	4.89	416
Parahippocampal gyrus	24 –48 –10	3.95	137
Precentral gyrus	–50 –10 26	4.12	131
Precuneus	–26 –78 18	4.03	152
Superior temporal gyrus	50 14 –10	4.22	189

See Figure 4 for visualization of the results.

**Figure 5:**
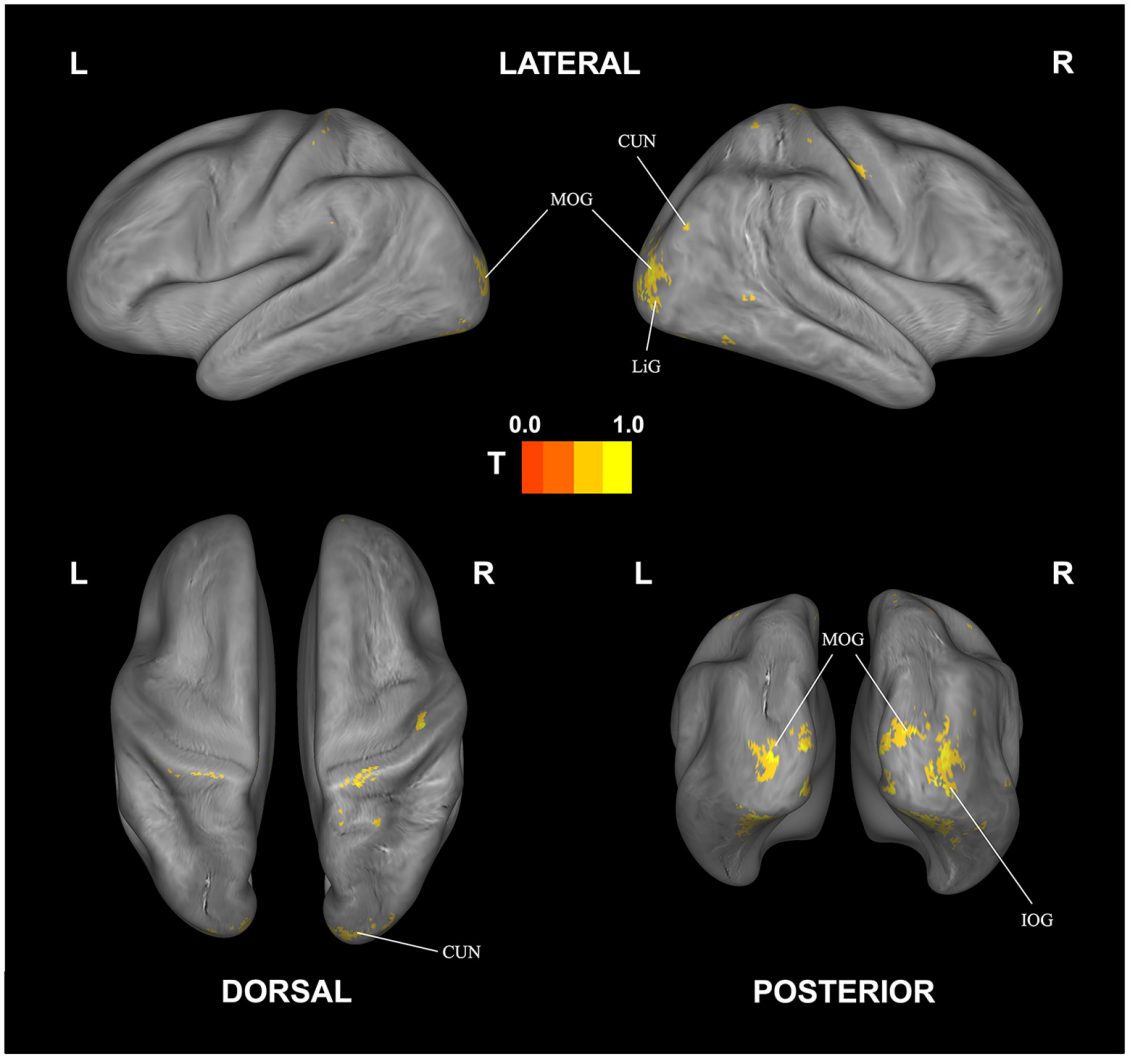
Correlational searchlight analysis results for subjective difficulty. The anatomical labels are abbreviated as follows: CUN, cuneus; IOG, inferior occipital gyrus; LiG, lingual gyrus; MOG, middle occipital gyrus. Result was FDR corrected (*p* < 0.05).

The corresponding decoding results for difficulty are reported in Table 4 and Figure 5. Similar to and overlapping with fatigue, we find that difficulty can be decoded in visual association areas, including the cuneus, declive, lingual gyrus, and the middle occipital gyrus. In addition, we find separate, selective activation in the inferior occipital gyrus.

**Table 4:** Searchlight analysis results detailing statistics of the significant regions for subjective difficulty (*p* < 0.05, FDR-corrected, >100 voxels).

**Region (AAL)**	**Peak voxel**	*Z* **-score**	**Number of voxels**
Cuneus	–8 –96 14	5.63	378
Declive	26 –68 –18	4.40	238
Inferior occipital gyrus	–26 –88 –14	5.35	132
Lingual gyrus	–16 –82 –8	6.04	1,069
Middle occipital gyrus	–26 –86 –14	5.17	594

See Figure 5 for visualization of the results.

**Figure 6:**
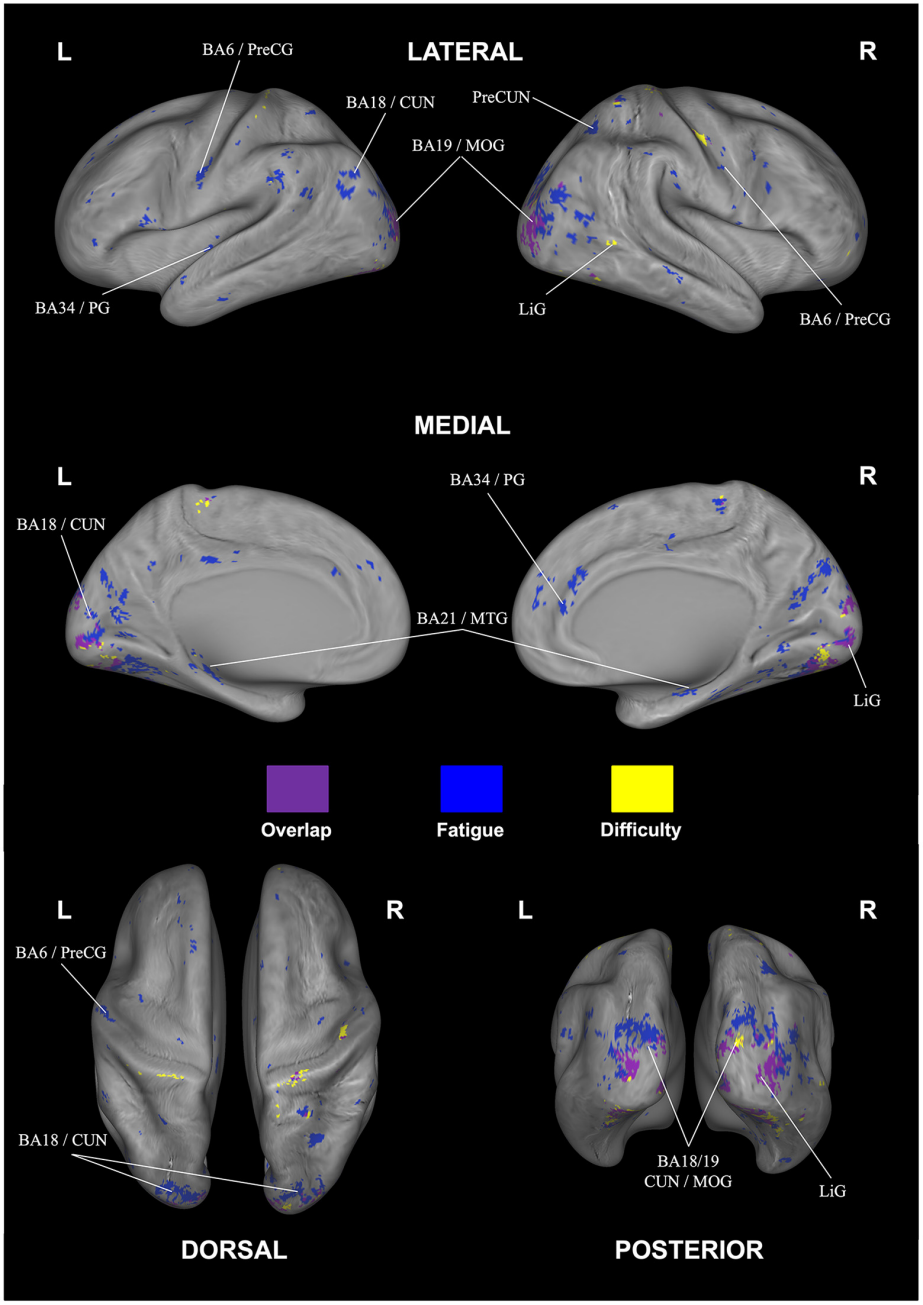
Overlap (purple) and rating-specific decoding for fatigue (blue) and difficulty (yellow). The anatomical labels are abbreviated as follows: CUN, cuneus; LiG, lingual gyrus; MOG, middle occipital gyrus; MTG, middle temporal gyrus; PG, parahippocampal gyrus; PreCG, precentral gyrus; PreCUN, precuneus. Results were FDR corrected (*p* < 0.05).

Looking at overlapping and individually selective areas in more detail, we uncovered similar, significant activation for subjective fatigue *and* difficulty in the cuneus, declive, lingual gyrus, and middle occipital gyrus. In addition, subjective fatigue on its own was decoded in parts of the culmen, middle occipital and middle temporal gyri, parahippocampal gyrus, precentral gyrus, and precuneus, whereas subjective difficulty had significant loading in parts of lingual gyrus (see Table 5, Figure 6).

**Table 5:** Searchlight analysis results detailing statistics of the significant regions for overlapping and individually selective areas following peak *Z*-score averaging and voxel thresholding (*p* < 0.05 FDR-corrected, minimum 100 voxels).

**Region (AAL)**	**Peak voxel**	*Z* **-score**	**Number of voxels**
Cuneus	–16 –94 6	5.38	271
Declive	26 –68 –20	4.29	135
Lingual gyrus	22 –84 –8	4.75	536
Middle occipital gyrus	–20 –94 10	5.09	422
Culmen	32 –58 –28	4.46	156
Cuneus	8 –90 24	4.51	689
Declive	–34 –60 –28	4.65	472
Lingual gyrus	–22 –76 –4	4.33	355
Middle occipital gyrus	26 –80 4	4.51	296
Middle temporal gyrus	42 –70 16	4.89	350
Parahippocampal gyrus	34 –22 –20	3.72	123
Precentral gyrus	–50 –10 26	4.12	107
Precuneus	–26 –78 18	4.03	144
Lingual gyrus	–18 –88 –12	5.29	217

See Figure 6 for visualization of the results.

### 3.4 Decoding functional correlates of subjective fatigue and difficulty

**Figure 7:**
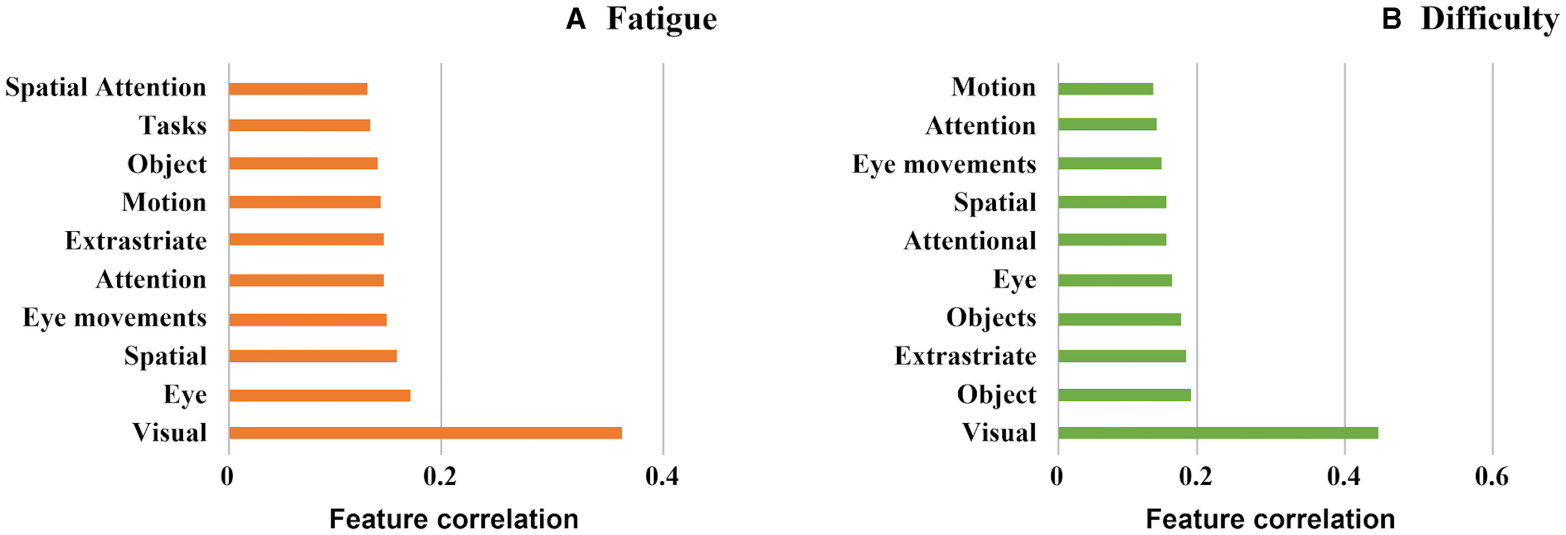
Associated psychological features using the decode function of neurosynth (Yarkoni et al., 2011) **(A)** Subjective fatigue **(B)** Subjective difficulty.

The decoding function of Neurosynth correlates the resulting activation patterns from our searchlight analyses against templates obtained from an automatic literature analysis of different key terms in neuroscientific research. Figure 7 shows the correlation strength for the 10 highest-correlating terms for the two activation patterns shown in Figures 4, 5 in ascending order. Overall, the strongest terms for both cases was the term “visual,” matching the strong involvement of visual areas we identified earlier. The nine terms listed next all appear at similar correlation strengths and focus both on eye movement and attentional processes—both crucial components of our visual search task. As the decoding analysis operates at whole-brain scale, the rank-ordering of these nine terms at our observed correlation strengths cannot be precise, and hence this analysis overall provides a global picture of the similarity of the two tasks at the whole network level.

## 4 Discussion

The aim of the present study was to decode brain-wide associations of subjective, visual fatigue as well as perceived difficulty facilitated through modulation of fatigue via myopia-correcting lenses and of difficulty via varying search task contexts.

In doing so, we first made sure that lenses were correctly fitted and participants were suitably adapted: in addition to following lens prescription guidance, the prescription strengths of both lens types were made identical to minimize displacement due to differences in lens power (Zheng et al., 2021; Zhen et al., 2022). Furthermore, to ensure that participants were adapted to both lens types, the experiment was conducted only after successful adaptation to each type of lens (SV and DIMS) was verified.

### 4.1 Behavioral results

On the behavioral side, we showed that while the DIMS lenses neither impacted accuracy nor response time, we replicated our most important previous finding (Ryu et al., 2021) with a significant reduction of subjective fatigue while wearing DIMS lenses compared to the SV lenses. The precise reason for why the DIMS lenses cause lower fatigue in our task context remains an open issue (Ryu et al., 2021). When exposed to a cognitive burden for an extended length of time, people often exhibit time-on-task (TOT) effects, in which performance deteriorates gradually with the duration of task engagement (Lim et al., 2010). The resulting subjective mental fatigue, however, may be reduced by modulating muscle stress (Thorud et al., 2012; Kollbaum et al., 2019; Kajita et al., 2020), via lower attentional load from the blurred peripheral input (via interactions between attention and defocus blur as shown in (De Lestrange-Anginieur et al., 2021), for example), or even via enhanced ocular blood perfusion (Leske, 2009)—further studies are necessary from an ophthalmological perspective in this context. In addition to this main effect, however, we also identified several correlations between measures: As may be expected, subjective fatigue and perceived difficulty were positively correlated—this result is in line with previous studies that have shown that increasing task difficulty plays an important role in mental fatigue (Bafna et al., 2021). In addition, accuracy and response time were affected by perceived difficulty levels—again, these are well-documented effects in visual tasks (Stone and Kadous, 1997; Goldhammer et al., 2014; Rath et al., 2015).

### 4.2 Imaging results

We next used whole-brain correlational searchlight analysis to investigate the association between neural activity and subjective fatigue and difficulty. As the overlapping and individually-selective brain areas shared common substrates, we first discuss areas in general that showed decoding for both fatigue and difficulty—theses include the cuneus, lingual gyrus, middle occipital gyrus (MOG), and declive.

The cuneus is known to receive and process visual information from the retina (Wyble and Swan, 2015; Gerard-Mercier et al., 2016; Yu et al., 2020)—more specifically, in the context of our experimental paradigm, previous studies (Bokde et al., 2006; Saur et al., 2010; Ranchet et al., 2017) indicated a positive correlation between cognitive task performance and cuneus activation modulated by the difficulty levels of task. Furthermore, studies of cortical-striate networks in cognitive tasks by Ren et al. (2019) and Wang et al. (2019) identified the cuneus (as well as posterior cingulate cortex and occipital gyrus) as correlating with cognitive load (visual load) of visual information processing in a challenging task—a result also corroborated in EEG studies (Lee et al., 2019) that showed correlations in brain activity in cuneus with language difficulty levels. Since our visual search task included sustained visual attention, continued spatial updating, and high working memory load, the involvement of the core visual processing areas for difficulty was to be expected—potentially as a result of the behavioral correlations connecting difficulty and fatigue, also subjectively-perceived fatigue would be affected. We also observed, however, fatigue-specific parts of the cuneus in our decoding. Since to our knowledge no other comparable fMRI study has directly implicated this brain area, support for this finding may come from previous EEG studies that have shown changes in alpha wave responses traceable to the cuneus correlating with changes in fatigue levels (Jung et al., 2013; Kim et al., 2019).

Lingual gyrus is another area highlighted in our analysis capable of decoding both difficulty and fatigue. This area is known to be activated during face memorizing, as well as maintaining, and controlling selective visual attention as a “node” or “hub” of visual information processing in complex image encoding (McCarthy et al., 1999; Kozlovskiy et al., 2014). Lingual gyrus, for example, was shown to play a vital role in encoding target face images (Taylor et al., 2009; Batouli et al., 2020), which is important in our visual search context, as participants need to remember Wally's face and clothing, while avoiding confusion with similar-looking distractor characters—all while maintaining and distributing attention throughout the search in the image. Since the Wally figure itself was more or less unfamiliar to participants, another potential connection comes from Demorest et al. (2010), who found greater lingual gyrus activation for unfamiliar compared to familiar stimuli. Again, parts of lingual gyrus were also able to only decode fatigue—as shown by Wylie et al. (2017a), this could be due to the strong memory aspects that have implicated lingual gyrus during a visual n-back task.

Next, middle occipital gyrus (MOG) was shown as a significant area of overlap for decoding fatigue and difficulty—this area also is known for its important role in visual attention, visual processing, and cognitive control and hence has prior support for its involvement in our task. Concerning fatigue, a prior clinical study showed an interaction between fatigue and behavioral responses of participants in the healthy control groups in MOG (as well as insula, precentral gyrus, thalamus, and culmen)—a result explained by the employed task-switching (Suda et al., 2009; Genova et al., 2013). We also note that the aforementioned study used younger participants similar to our paradigm; in this context, Ansado et al. (2012) and Maximo et al. (2016) showed increased activation of MOG during visual selective attention tasks—additional experiments would need to done to investigate the degree to which MOG may still be clearly implicated in adults. Concerning difficulty, an older study by Gerlach et al. (1999) showed correlations of brain activity in MOG with difficulty during visual form matching. This was extended also to visual spatial processing tasks in Kesler et al. (2004) and Zarnhofer et al. (2013).

The final area of overlap in decoding was the declive. This area has been implicated in a prior study on fatigue within a high-difficulty task (along with vermis and precentral gyrus) especially during memory retention—hence more effort to encode, maintain, and retrieve information in memory seemed to go along with increased mental fatigue (Charroud et al., 2015). The declive was also highlighted in the previously-mentioned study on cognitive fatigue resulting from two different levels of N-back tasks (Wylie et al., 2017b). Again, however, we found fatigue-selective decoding in parts of this area—one potential source of this may be the involvement of the declive in coordination of eye movements associated with task-level control and focal attention (Parker et al., 2014)—both core requirements for solving the visual search task.

Next, we discuss the areas implicated in decoding of fatigue—this includes five additional brain regions (culmen, parahippocampal gyrus, precentral gyrus, middle temporal gyrus, and precuneus). The culmen, which plays a role in cognitive and emotional processing as well as in movement and fine motor control, was implicated as a region of interest in a previous clinical study on chronic fatigue (Barnden et al., 2019)—specifically, healthy controls possessed greater connectivity in this area in relationship to their performance in cognitive tasks (see also Wu et al., 2013). In addition, Egner and Hirsch (2005) showed functionally increased interactions between the culmen, superior temporal gyrus (SFG), anterior lobe, and cerebellum in the conflict Stroop task. Similarly, Simmonds et al. (2007) showed that the culmen was associated with task performance in both Go/No-go tasks for children—in this case, potentially related to the circuits involved in preparing motor responses. The activities in these tasks share critical aspects with the demands of our visual search task in which competing hypotheses for the location of the target need to be continuously tested and updated during the task duration.

The parahippocampal gyrus was implicated in a previous, older study on the Stroop task as well (Krabbendam et al., 2000), potentially as the parahippocampal area serves as a network of connections to the dorsolateral prefrontal, temporal and limbic areas to maintain task-related information. N-back task studies specifically investigating mental fatigue with EEG and MEG Shigihara et al. (2013) showed alpha power increases in the parahippocampal gyrus as part of the thalamo-frontal feedback loop—results corroborated in an fMRI study by Gavelin et al. (2017). In the latter study, the task-resulting fatigue was partially explained as a reward mechanism recruiting additional cognitive resources to sustain task performance as more demands in terms of cognitive control (Nee, 2021) were placed on the brain. Again, this aspect of cognitive control fits well into the context of the sustained visual search task demands in the present study.

Concerning the implication of the precentral gyrus (PCG) in fatigue, recent research using fMRI by Batouli et al. (2020) studying mental fatigue in facial or word memory tasks implicated PCG—of note in particular in our context would be the relevance of the face memory aspect. Another study (Genova et al., 2013) evaluated neural correlates of cognitive fatigue in a multitasking also highlighting the relationship of PCG with fatigue levels— in that study specifically referred to as 'state fatigue' while performing cognitive tasks. Furthermore, this type of fatigue from cognitive tasks has been linked to increased connectivity between the PCG and hippocampal regions (Stefancin et al., 2019). Turning to the middle temporal gyrus (MTG)—a region known for memory and higher-level information processing—the aforementioned EEG studies discussed in Shigihara et al. (2013) showed increased power for two types of tasks, suggesting that this area may be related to task performance during the fatigue-inducing test. Beyond this, a clinical study on fatigue levels in Parkinson's patients (Cho et al., 2017; Zhang et al., 2018) showed correlations of the Fatigue Severity Scale (FSS) with MTG activation.

Finally, we discuss the involvement of the precuneus in decoding of fatigue. A recent study by Fandakova et al. (2021) and Tomasino et al. (2022) implicated the precuneus (together with IFG) in mental fatigue during visual imagery tasks in retrieval and recall conditions. Interestingly, connectivity of the precuneus with thalamic/striatal areas was also modulated with memory-related performance in an auditory task (Paced Auditory Serial Addition Test; PASAT—see (Boissoneault et al., 2018). Additionally, the precuneus was also highlighted as involved in mental fatigue in the previously-discussed studies in different cognitive tasks (Wylie et al., 2017a; Bruijel et al., 2022). Overall, given the strong memory aspect of the visual search task in the present study, the involvement of the precuneus seems well-founded.

Although our context was visual fatigue as assessed by the in-scanner questionnaire, we found decodability of the fatigue levels (different from decoding of perceived difficulty) in a wide-spread network of brain areas. As pre-existing studies on visual fatigue related to eyestrain directly are relatively rare, comparison with prior research is limited. A study by Kang et al. (2021) showed that different types of refractive errors (myopia and hyperopia) can alter activation patterns of the brain; specifically, compared to a control group, individuals with myopia and hyperopia exhibited higher levels of brain activation when processing visual stimuli (myopic defocus = right precentral gyrus, right superior temporal gyrus, left inferior parietal lobule, and left middle temporal gyrus; hyperopic defocus = right and left precentral gyrus). Hence, it will be interesting to extend our study to participants with normal vision to test generalizability of our results. In addition, one study using Near-Infrared-Spectroscopy (NIRS) highlighted increased oxygenation in pre-frontal cortex due to visual fatigue (Richter et al., 2018). This part of the brain was not implicated in our study, with results focused on other brain networks. The direct comparison of NIRS responses and fMRI responses is difficult, however. Similarly, the study assessing brain activity under stereoscopic viewing conditions (Chen et al., 2017) suggested that increased interaction in visual areas BA18/19 may be associated with the increased visual fatigue during stereoscopic viewing. These results are well in line with our findings, however, our study reveals a wider-reaching range of brain areas beyond the interactions of BA18/19 regions associated with fatigue reduction.

Interestingly, many of the regions highlighted in our decoding fit with the results from prior studies working with cognitive fatigue as discussed above. Specifically, results from studies dealing with discrete processing tasks, in which stimuli are presented and processed independently, and participants are required to make a response to each stimulus before moving on to the next one. These tasks include our visual search paradigm and require focused attention on each individual stimulus known to induce cognitive fatigue already at relatively short durations of 30 min (Breckel et al., 2013; Sun et al., 2014; Borragán et al., 2017; Malik and Amin, 2017). Overall, we suggest that our results therefore relate both to the lower-level aspects of visual fatigue as well as its higher-level, associative effects.

### 4.3 Adolescent sample

As stated in the introduction, the present study focused first on exploring the neurological underpinnings of visual fatigue in an adolescent sample, given that these are most at risk of impairments due to myopia progression (Baird et al., 2020) and also are among the age groups with the highest exposure to digital screens (Kim et al., 2017). This represents a potential, two-fold limitation of the present study in terms of generalizability due the presence of myopia and the restricted age range.

Concerning the effect of myopia, prior research has shown a consistent *positive* correlation in adolescents between cognitive function and myopia (Megreli et al., 2020) in particular with verbal intelligence. Although there is no data available on how this would impact, for example, visual search skills, our sample of myopic adolescents may be slightly skewed toward better cognitive function because of this, highlighting the need for future studies with other samples. Concerning the effect of age, the period from adolescence to adulthood is known to introduce significant brain changes (Couperus and Quirk, 2015), including structural and functional changes such as myelination and alterations in neurotransmitter systems like dopamine and serotonin. The differences between adults and children are not limited to biological factors but also extend to the functional aspect of selective attention processing in the visual search. Taken together, it is conceivable that those changes also may have an impact on the task performance as well as on the experience of fatigue. Given their weaker selective attention compared to adults, children may, for example, struggle to effectively suppress task-related characteristics, leading to a more pronounced degree of fatigue caused by relatively strong attention (Frank et al., 2021). In our previous, behavioral study (Ryu et al., 2021) we found differences in terms of task performance (accuracy and response time) for adults vs. adolescents, however, the overall effect of the DIMS lenses on perceived fatigue was similar in both samples. Again, further tests with an adult sample are needed to investigate the degree to which the results will generalize across a larger age range.

## 5 Conclusion

The present study is to our knowledge the first study attempting to decode fatigue vs. difficulty by means of external modulation of fatigue via myopia-correcting lenses. Our multivariate representational similarity analysis highlights several brain regions correlating with both subjectively perceived fatigue and difficulty. Beyond these, importantly, we also implicate additional brain regions that seem to be more specific to decoding of fatigue only. Overall, these regions indicate a network of areas encompassing the full range from lower-level processes such as visual processing, spatial attention, working memory, and face memory to higher-level cognitive control. This network fits well with prior knowledge, but crucially is able to dissociate aspects of subjectively perceived difficulty from those of subjectively-perceived fatigue.

In addition to extending the age sample to include adults, visual fatigue and its associated, higher-level effects will need to be investigated using a wider battery of tasks. Similarly, so far it is not known whether the DIMS lenses employed here sustain their fatigue-reducing effect over a longer period of time, leaving room for further studies. Likewise, our focus here was in using the lenses as a means to decode fatigue (vs. difficulty); we have tried to decode lens type as well, but since this analysis can only use half of the trials (10), the power for this analysis is lower compared to the decoding analyses of fatigue and difficulty—hence, in order to provide a reliable decoding of lens type, we would need to run more trials. Finally, other methodological approaches such as functional and anatomical connectivity analyses could be used to augment the present results.

## Data availability statement

The datasets presented in this study can be found in online repositories. The names of the repository/repositories and accession number(s) can be found at: https://osf.io/32s7d/?view_only=726938922ebe4abfae1106d7f791e016.

## Ethics statement

The studies involving humans were approved by Internal Review Board of Korea University (KUIRB-2019-0310-06). The studies were conducted in accordance with the local legislation and institutional requirements. Written informed consent for participation in this study was provided by the participants' legal guardians/next of kin.

## Author contributions

HR: Conceptualization, Formal analysis, Investigation, Resources, Writing – original draft. UJ: Formal analysis, Investigation, Validation, Writing – review & editing. CW: Formal analysis, Funding acquisition, Project administration, Supervision, Writing – review & editing.
