# Supplementary material for: Decoding visual fatigue in a visual search task selectively manipulated via myopia-correcting lenses
Source: Front Neurosci. 2024 Apr 10;18:1307688. doi: 10.3389/fnins.2024.1307688 (PMC11039808; doi:10.3389/fnins.2024.1307688)
Supplement: Supplementary file 1 [file Data_Sheet_1.PDF]

Supplementary Material

1 SUPPLEMENTARY DATA

2 SUPPLEMENTARY TABLES AND FIGURES

2.1 Tables

Table S1. Location information of the correlational searchlight analysis for subjective fatigue. All analyses are p<.05 FDR corrected. **Supplement Figure** S1 for visualization of the results.

Region(AAL)	Peak Voxel	Z-score	Number of voxels
Anterior Cingulate	-34 -72 32	4.12	110
Cingulate Gyrus	-2 -22 36	4.20	379
Culmen	32 -54 -22	5.03	471
Cuneus	-22 -88 8	6.40	2029
Declive	-20 -78 -18	6.19	1005
Fusiform Gyrus	-32 -82 -18	6.11	722
Inferior Frontal Gyrus	42 6 36	4.55	474
Inferior Occipital Gyrus	-30 -86 -16	5.70	192
Inferior Parietal Lobule	-36 -54 56	4.91	1349
Insula	58 -32 20	4.23	162
Lingual Gyrus	-18 -82 -12	6.56	1389
Medial Frontal Gyrus	-2 -2 60	4.44	661
Middle Frontal Gyrus	-26 52 22	4.55	1228
Middle Occipital Gyrus	-20 -90 10	6.08	1202
Middle Temporal Gyrus	40 -74 16	4.65	1139
Paracentral Lobule	6 -44 58	3.98	146
Parahippocampa Gyrus	24 -54 -8	4.51	233
Postcentral Gyrus	-34 -34 64	5.01	1157
Posterior Cingulate	-18 -62 12	4.41	305
Precentral Gyrus	-50 -6 6	4.33	572
Precuneus	-32 -74 34	5.60	2106
Superior Frontal Gyrus	8 12 58	4.35	541
Superior Parietal Lobule	-32 -78 26	5.33	717
Superior Temporal Gyrus	-26 -58 44	4.56	765
Supramarginal Gyrus	-54 -52 16	4.32	183

2.2 Figures

Table S2. Location information of the correlational searchlight analysis for subjective difficulty. All analyses are p<.05 FDR corrected. See **Supplement Figure** S2 for visualization of the results.

Region(AAL)	Peak Voxel	Z-score	Number of voxels
Angular Gyrus	-48 -64 32	4.30	112
Cingulate Gyrus	-8 -42 34	4.27	254
Culmen	16 -68 -14	4.59	239
Cuneus	-24 -92 8	6.10	1662
Declive	-34 -60 -18	4.88	512
Fusiform Gyrus	-38 -46 -18	5.25	731
Inferior Frontal Gyrus	42 6 34	4.92	357
Inferior Occipital Gyrus	34 -82 -8	5.82	192
Inferior Parietal Lobule	-34 -58 42	5.01	930
Inferior Temporal Gyrus	38 -74 -2	4.60	107
Lingual Gyrus	22 -88 -6	6.58	1400
Medial Frontal Gyrus	2 -4 58	4.88	427
Middle Frontal Gyrus	34 -4 48	4.85	581
Middle Occipital Gyrus	10 -94 10	6.59	1436
Middle Temporal Gyrus	-46 -70 10	4.94	1128
Parahippocampa Gyrus	-28 -50 -10	4.44	259
Postcentral Gyrus	-44 -36 54	5.32	589
Posterior Cingulate	-8 -68 8	3.68	139
Precentral Gyrus	36 -4 46	4.26	289
Precuneus	-28 -54 50	5.22	1521
Superior Frontal Gyrus	4 10 54	4.67	304
Superior Parietal Lobule	-24 -64 56	5.10	789
Superior Temporal Gyrus	-52 -52 14	4.51	578

Table S3. Location information of the correlational searchlight analysis for subjective fatigue. All analyses are p<.05 FWE corrected. See supplement figure S5 for visualization results.

Region(AAL)	Peak Voxel	Z-score	Number of voxels
Cuneus	-16 -94 6	5.46	10
Middle Occipital Gyrus	28 -84 4	5.53	18

Table S4. Location information of the correlational searchlight analysis for subjective difficulty. All analyses are p<.05 FWE corrected. See supplement figure S6 for visualization results.

Region(AAL)	Peak Voxel	Z-score	Number of voxels
Cuneus	-8 -94 14	5.63	14
Lingual Gyrus	-16 -82 -8	6.04	28
Middle Occipital Gyrus	-26 -86 -14	5.17	11

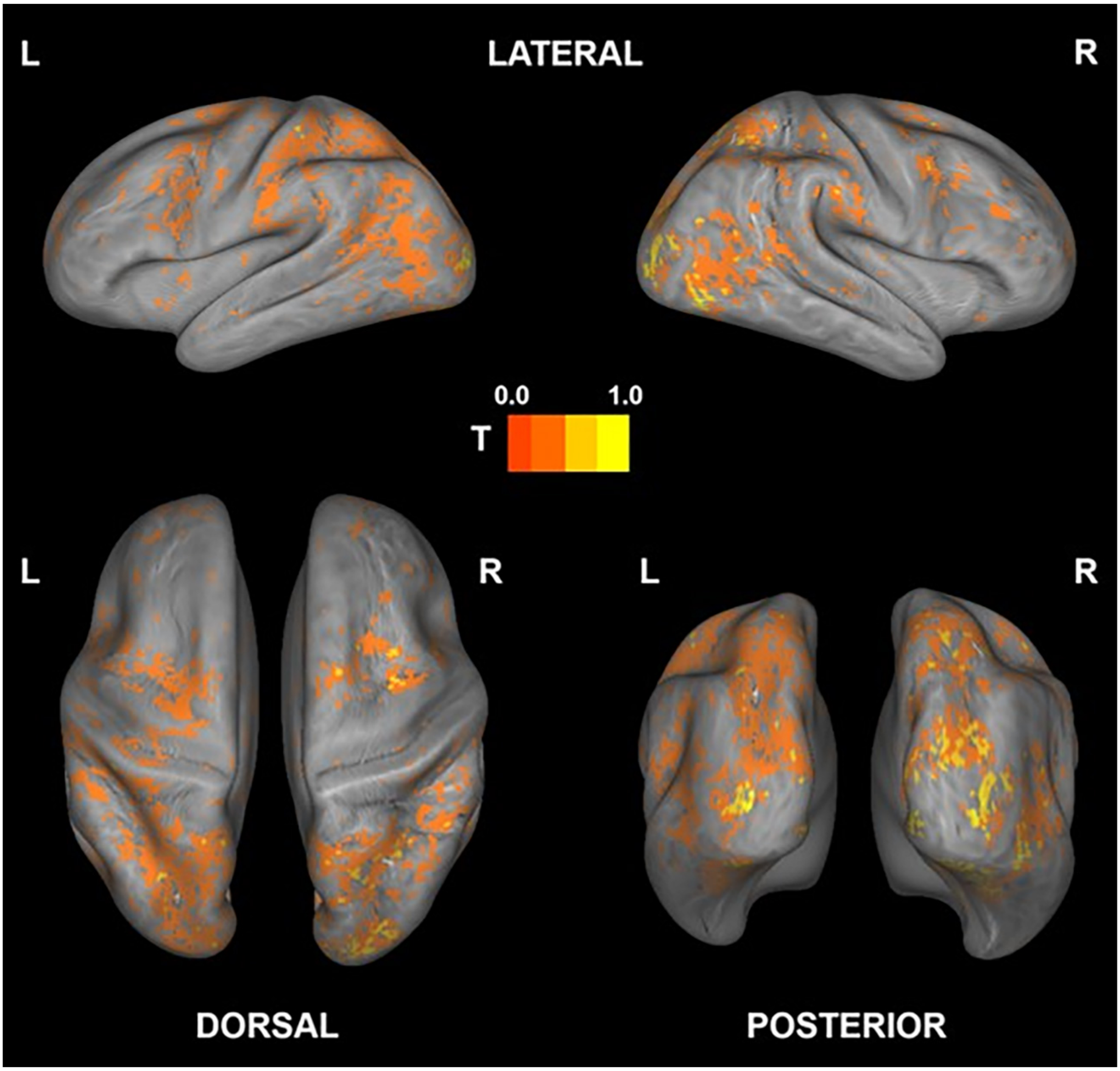

Figure S1. Correlational searchlight analysis results for subjective Fatigue. Result were FDR corrected ($p<.05$)

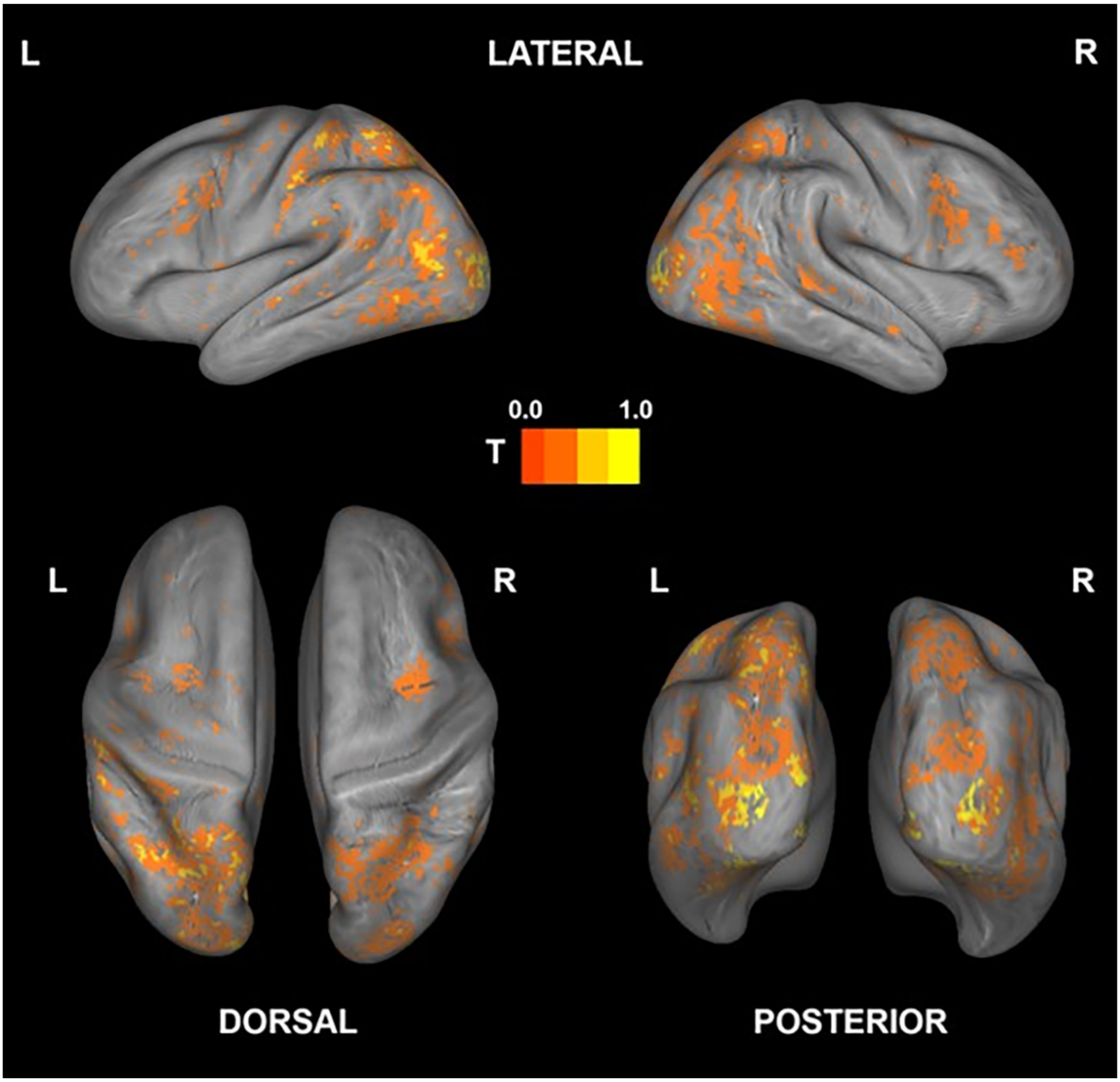

Figure S2. Correlational searchlight analysis results for subjective Difficulty. Result were FDR corrected (p<.05)

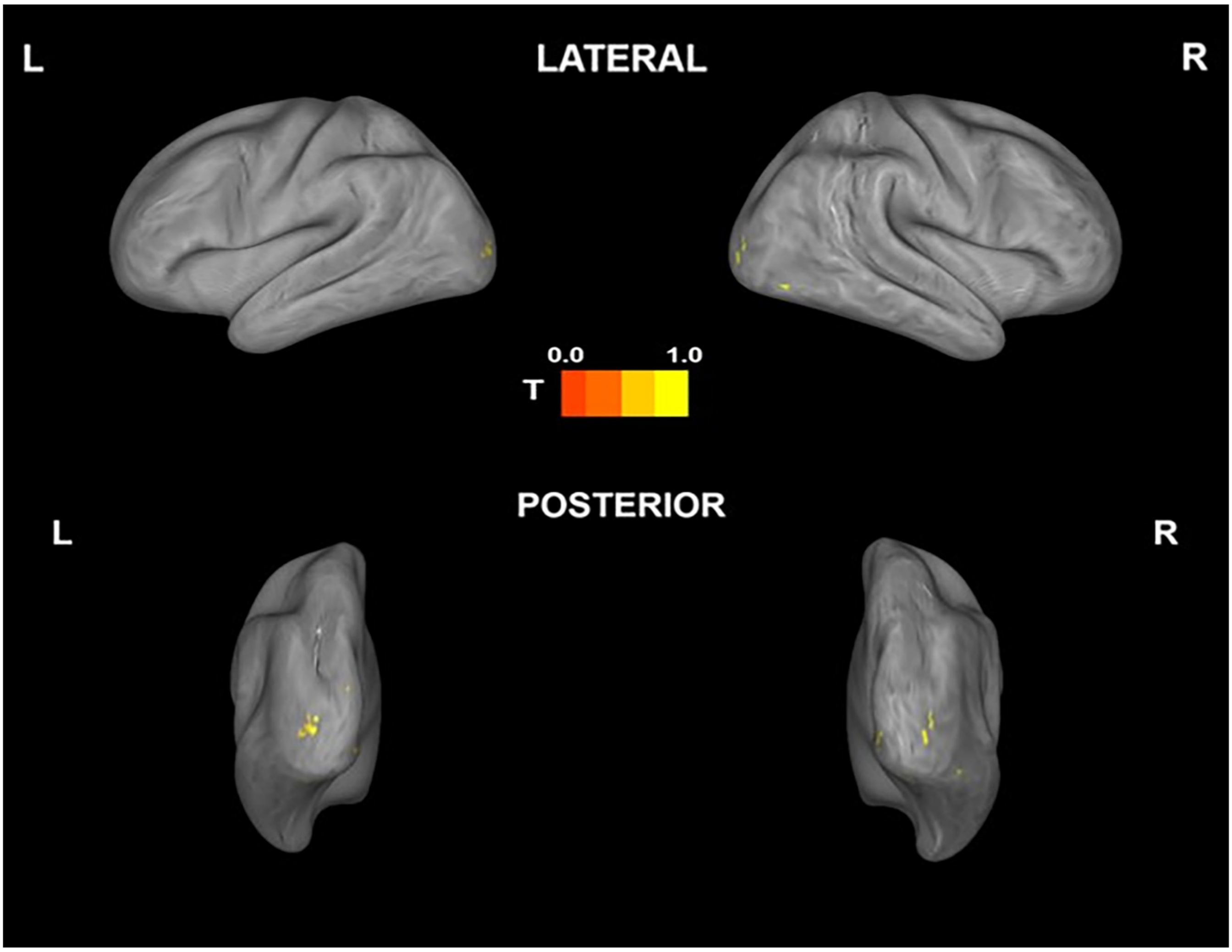

Figure S3. Correlational searchlight analysis results for subjective Fatigue. Result were FWE corrected (p<.05)

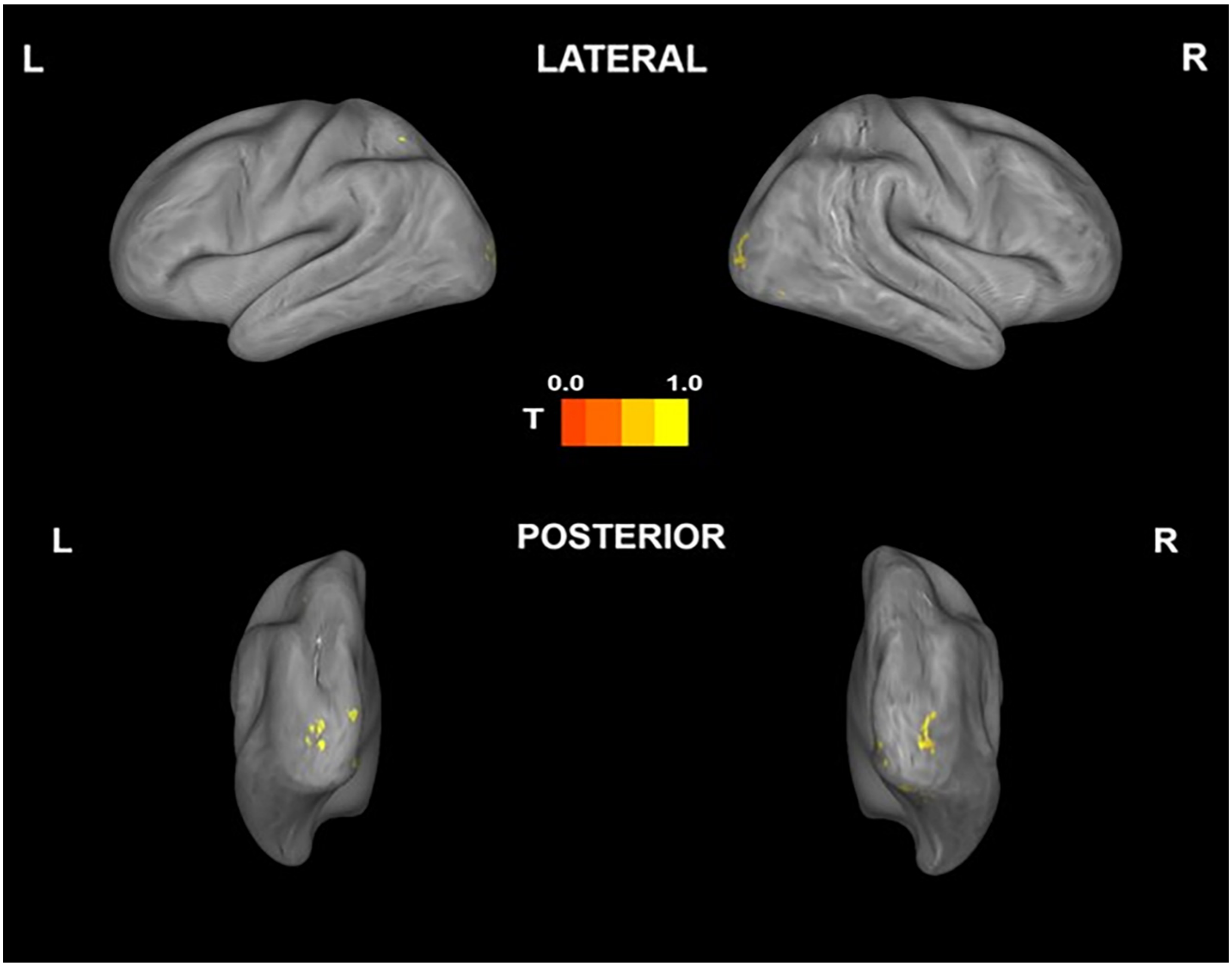

Figure S4. Correlational searchlight analysis results for subjective Difficulty. Result were FWE corrected (p<.05)

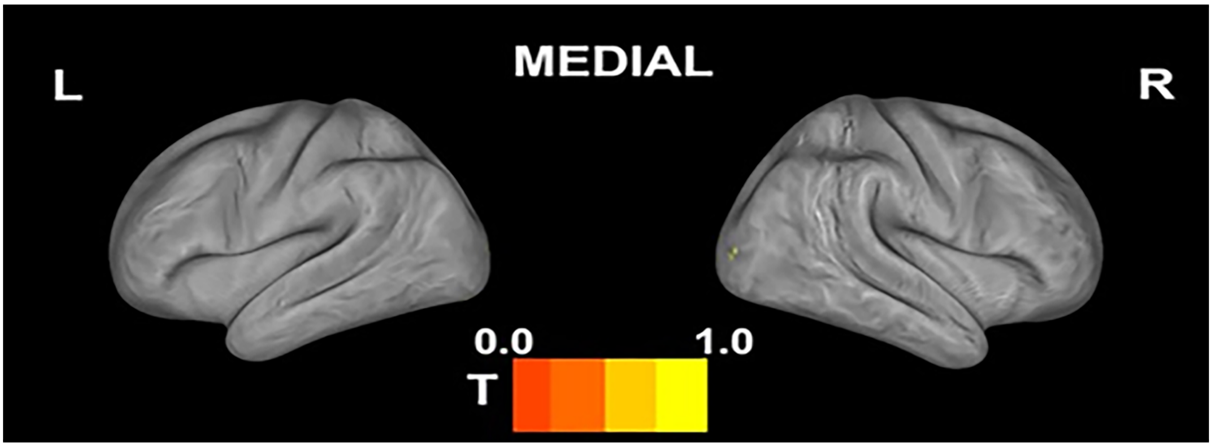

Figure S5. Correlational searchlight analysis results for subjective fatigue for individual images. Results were FWE corrected (p<.05)

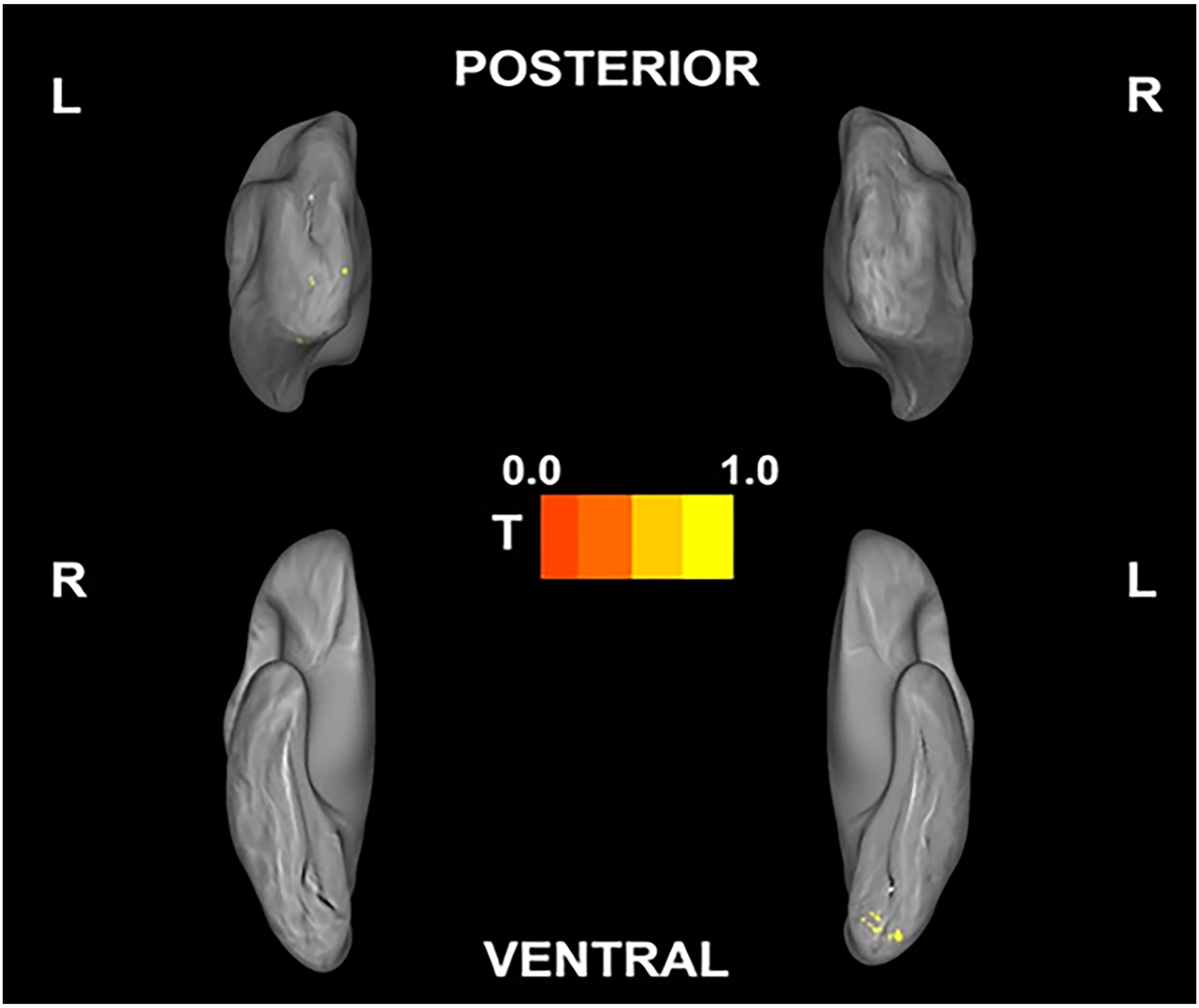

Figure S6. Correlational searchlight analysis results for subjective difficulty for individual images. Results were FWE corrected ($p<.05$)

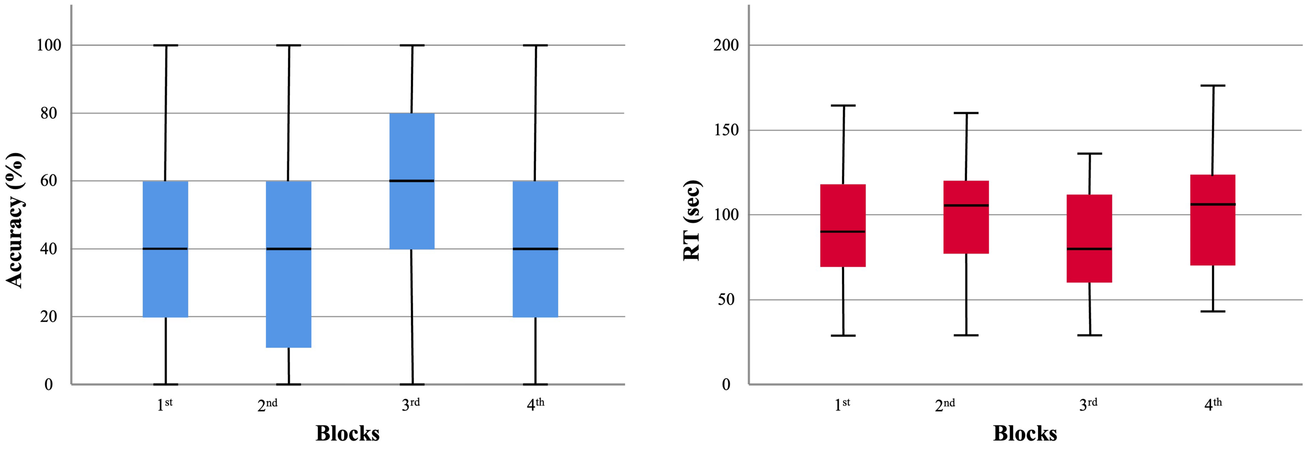

Figure S7. No significant changes by time progress were observed in accuracy(left) or reaction time (right) across blocks.
